# Forced displacements at the human–animal interface: a narrative review of knowledge gaps and One Health insights

**DOI:** 10.1093/inthealth/ihag038

**Published:** 2026-04-29

**Authors:** Giorgia Casti, Chandralekha Nair, Emanuele De Ponti, Francesca Squillace, Piera Ceschi

**Affiliations:** Independent Researcher, 09121 Cagliari (CA), Italy; Independent Researcher, 688001 Alappuzha, Kerala, India; University Vita-Salute San Raffaele, Faculty of Medicine, via Olgettina 58, 20132 Milan, Italy; Fondazione IRCCS Ca’ Granda Ospedale Maggiore Policlinico, via Francesco Sforza 28, 20122 Milan, Italy; South Tyrolean Healthcare Services, Department for Prevention, Teaching Hospital of Paracelsus Medical University, via Dante-Straße 51, 39042 Bressanone/Brixen (BZ), Italy

**Keywords:** displacement, migration routes, One Health, vulnerable populations, zoonoses

## Abstract

Environmental emergencies and conflicts are major drivers of human and animal displacements, increasing the spread of communicable diseases. In such contexts, livestock keepers are among the most vulnerable communities. As a matter of fact, when animals are moved, tensions between groups may escalate and disease spread can intensify. In contrast, when livestock are left behind, households face severe economic decline and increased poverty. This article explores the phenomenon of forced displacements at the human–animal interface, potential gaps and opportunities. This narrative review is based on a search of PubMed and grey literature. Peer-reviewed literature published from 2015 to March 2025 and reports and documents from governmental institutions, non-governmental organizations (NGOs) and press websites in English, French, German, Hindi, Italian, Portuguese and Spanish were considered eligible for analysis. Thirty-four peer-reviewed papers met the inclusion criteria. The majority of the studies were based in the African Continent on the main migration routes, addressing mainly vector-borne diseases, capacity-building needs and a lack of research. Grey literature was incorporated alongside peer-reviewed papers as governments, intergovernmental organizations, NGOs and the press represent relevant actors in implementing coping strategies and raising awareness on forced animal displacements. This narrative review highlights the need for primary data collection and interdisciplinary collaboration adopting a route-based and equity-oriented One Health approach.

## Introduction

Environmental emergencies and conflicts are major drivers of human displacements. A comprehensive understanding of the interconnections between migration patterns and the demographics of the affected populations is imperative to address the dynamics of environmental- and war-related forced displacements. Among the environmental variables, water represents one of the most critical factors, and populations in arid regions are most exposed to droughts and more likely to migrate.^1^

A forced displacement, whether due to environmental emergencies, natural disasters or conflict, has the potential to engender a situation in which both animals and their owners are displaced and become refugees. Although refugees are by definition people who have fled their countries to escape conflict, persecution, violence or natural disasters, these same factors can also lead to internal displacements, with consequences for both human and animal health. All these displacements need to be addressed. From a public health perspective, all these conditions may increase the risk of exposure to pathogens with zoonotic potential; however, this phenomenon can be underreported and underestimated.^2^

In such contexts, livestock keepers are among the most vulnerable communities. In the worst-case scenario, they have to abandon their animals, and this can result in the loss of valuable farm animals that are a crucial means of income and sustenance for their households.^3^ Importantly, when livestock keepers have to abandon or lose their livestock, their chances of returning home are reduced^4^ and entire communities are at risk of losing their means of subsistence and falling back into poverty.

In contrast, when animals are forced to move with their owners, disputes among communities may arise and the risk of transboundary diseases may increase. It is therefore vital to acknowledge the relevance of human–animal interconnections and the interactions among displaced communities with the local hosting populations in order to minimize displacement-related issues and to share solutions for a peaceful coexistence. To this end, the development of preparedness strategies, targeted agreements and joint policies on enhancing the resilience of these populations is imperative.^5^

Forced displacements of farm animals along with their owners have been explored at the Refugee Studies Centre of Oxford University and the results of seven articles have already been published.^6^ Working animals are often involved in relocation efforts, as they can facilitate human movements; however, their presence within or close to refugee camps requires the implementation of context-appropriate management strategies. The administration of the Sahrawi refugee camps in the Algerian desert provides a notable example on how agreements among local residents, nomadic populations and refugees can mitigate the impact of forced displacements during civil conflicts.^7^ A further valuable strategy—adopted to address climatic challenges in the Indian State Maharashtra—is the establishment of cattle camps during drought episodes. These camps represent a critical adaptation measure, mitigating the detrimental impact of extreme weather conditions by ensuring access to essential resources and providing care, allowing the survival of rural communities and thus safeguarding their livestock-dependent livelihoods.

The consequences of displacements may also be experienced by companion animals. Sometimes they cannot follow their owners, like the dogs in Chernobyl, which were left behind.^8^ In other situations they are permitted to escape with their owners. A significant example is the adoption of a Temporary Protection Directive by the European Union (EU) in response to the Ukrainian crisis in 2022. The measures taken included temporary permission of non-commercial introduction of pets from Ukraine to the Member States despite these animals not meeting the European Animal Health Law requirements.

Despite growing recognition of the One Health dimensions of displacement, there is no comprehensive synthesis of the health impacts, coping strategies and policy gaps related to forced animal displacement. This narrative review explores the documented forced displacements at the human–animal interface, highlighting conflict and disaster settings, to identify gaps in the peer-reviewed and grey literature and to advocate for a route-based, equity-oriented One Health framework for intervention.

## Methods

A preliminary search on Google Scholar was useful to describe the background and contextualize this narrative review, followed by an advanced search in PubMed using the same search terms. This database was selected because the primary focus of this search was on health-related literature, particularly human and animal health in humanitarian contexts, while PubMed offers comprehensive coverage of biomedical and global health research. Moreover, it provides access to peer-reviewed scientific literature without requiring institutional affiliation and it is free of charge, making it accessible to anyone. This ensures inclusivity and cost-effectiveness in the search process. Sources in English, French, German, Hindi, Italian, Portuguese and Spanish published from 2015 to March 2025 and focusing on displaced communities and refugees along with their animals or exposed to the risk of zoonoses, food-borne diseases and vector-borne diseases were considered eligible for analysis.

To compile the general reference list the following search items were used: ‘Animals OR livestock OR cattle OR goat OR sheep OR camelids OR equine OR birds OR dogs OR cats’ AND ‘refugees’ AND ‘camps’.

While the PubMed database was chosen for the literature search, the Rayyan data storage system (https://www.rayyan.ai/) was used to screen, store and sort references in a systematic way. This software made it possible to import references directly from the online database and share the results of the screening among the authors.

Importantly, the websites of the United Nations High Commissioner for Refugees (UNHCR), US Centers for Disease Control and Prevention (CDC), World Organisation for Animal Health (WOAH), International Organization for Migration (IOM), United Nations Children`s Fund (UNICEF), Food and Agriculture Organization (FAO), World Health Organization (WHO) and Red Cross were screened to identify relevant guidelines and documents suitable for inclusion in this article. These institutions provide authoritative data sources and normative frameworks directly pertinent to the subject matter.

Given the scarcity—or complete absence—of evidence concerning the specific context of the Indian cattle camps, official communications and documents issued by the Districts of Maharashtra were also examined and complemented with press reports to highlight existing knowledge and areas of ongoing debate. The research on this setting further incorporates information from non-governmental organizations (NGOs) active in humanitarian and development work, drawing on material published on their websites to integrate perspectives from relevant civil society actors engaged in these settings.

## Results

The research on PubMed gave 34 results and no duplicates were found. All the abstracts were read and after this first screening, all full-text manuscripts were read and assessed for eligibility. All 34 studies were considered eligible for inclusion in the review as they met the following inclusion criteria: full-text availability, publication date between 2015 and 2025, languages (English, French, German, Hindi, Italian, Portuguese and Spanish) and focus on displaced communities and refugees along with their animals or exposed to the risk of zoonoses, food-borne diseases or vector-borne diseases.

To summarize the critical appraisal of these papers a table (Table 1) was completed with the following items: reference (author, year), country, setting, aim of the study, zoonotic aspects and related issues, actions to manage the displacement.

**Table 1:** Papers from PubMed included in this review.

Author	Year	Country	Setting	Aim of the study	Zoonotic aspect and related issues	Actions to manage the displacement
Allan et al.^25^	2023	Yemen	Internally displaced people and hosting communities	To confirm the presence of *Anopheles stephensi* among displaced people and host communities	The growing number of conflicts causes unplanned population displacement into temporary camps. Increased urban malaria is a major threat. Populations depend on open water containers, which are favourable breeding sites for vectors	Proactive monitoring and targeted vector management
Al-Sanouri et al.^26^	2023	Jordan	Refugee camps	Seroprevalence of human brucellosis in Syrian refugees	Having animal-related occupations, residing outside refugees’ camps, consuming unpasteurized milk, handling animals or their tissues, and slaughtering animals within six months of study inclusion are factors significantly higher among the seropositive group.	Control measures: vaccination of the animals, implementation of laboratory detection and surveillance capacities, safe use and preparation of dairy products and safety practices of handling animals and their tissues
Azizi et al.^27^	2022	Tanzania	Refugee camps	Efficacy of blankets treated with permethrin and piperonyl butoxide in refugee camps	New vector control strategy for malaria vector control in refugee camps, where indoor residual spraying and insecticidal nests cannot be used	Impact of the intervention and acceptability study
Bagulo^28^	2021	Sub-Saharan Africa	Refugee and internally displaced person camps	Systematic review to investigate hepatitis E virus epidemiology in Africa	Conflicts causing displacements are associated with 50% of reported outbreaks of hepatitis E	Further research to investigate sources of contamination and transmission routes
Beiras et al.^29^	2025	Congo, Nigeria	Refugee camps	Investigation into concurrent outbreaks of monkeypox	For some clades, zoonotic transmission played an important role. The outbreak in the Democratic Republic of Congo has now expanded to several other countries and is spreading through direct and sexual contact in urban centres and refugee camps	Strong public health responses in terms of improved access to testing in remote areas and vaccination
Biswas et al.^30^	2021	Bangladesh	Host communities of Rohingya camps	Analysis about residential satisfaction	Livestock and agricultural land losses are factors associated with dissatisfaction	Policies and programs for the host communities involving both governments and NGOs
Brechot et al.^31^	2018	Jordan	Syrian refugees	Accomplishment of the Global Virus Network reported during the International Meeting in 2018	Respiratory infections are among the leading causes of mortality among refugee populations	To rationalize the use of new vaccines among displaced people
Chambers et al.^32^	2018	Syria	Refugees and displaced populations	Identification of *Phlebotomus papatasi* habitats	Refugees and internally displaced people are commonly in areas characterized by the presence of fly habitats, potentially leading to higher prominence of *Leishmania* infections	Stronger medical relief
Claus et al.^33^	2018	France	Refugee camp in Calais, France	Strategies to control roundworms (*Ascaris lumbricoides*)	Prevalence of ascariasis in industrialized countries is increasing because of immigration and the increasing number of refugees. Access and follow-up to monitor the implementation of hygiene measures are expensive and very difficult to organize in refugee camps, even in wealthy, industrialized countries	Measure required: periodic mass drug administration and water, sanitation and hygiene
Deng et al.^34^	2023	Algeria, Burundi, Rwanda and East Africa	Jails, homeless population and Niger and East African refugees	Comprehensive review and update on louse-borne infectious diseases/bacterial pathogens	Human lice have always been a major public health concern due to their vector capacity for louse-borne infectious diseases, like trench fever, louse-borne relapsing fever and epidemic fever, caused by *Bartonella quintana, Borrelia recurrentis* and *Rickettsia prowazekii*, respectively	Control measures: improved hygiene measures and delousing
Elzain et al.^35^	2025	Sudan	Refugee camps	*Schistosoma haematobium*	No infection was observed among residents of refugee camps	Control and monitoring of urogenital schistosomiasis among the local population
Fayemi et al.^36^	2018	Jordan and Cambodia	Internally displaced people	Tackling food insecurity	Animal by-products can be used	Diet-based interventions
Fotakis et al.^37^	2019	Greece	Refugee camps	Identification of *Leishmania* species in sandflies	The pathogen has been detected and the risk of infection is increasing in these communities	Measures: detection and treatment of leishmaniasis, community education and vector control
Fotakis et al.^38^	2020	Greece	Refugee camps	Evaluate the risk for vector-borne disease transmission within refugee communities camps in Greece	Disease vectors and pathogens in vectors were recorded in the refugee camps, indicating a situational risk factor for disease transmission	Targeted actions ensuring adequate living conditions and the establishment of integrated vector-borne disease surveillance programs
Gabaldon et al.^39^	2023	Ethiopia, Uganda and Pakistan	Refugees	This review aims to measure the effectiveness of topical repellents to prevent malaria infections	The use of topical repellents may be particularly useful for populations who may not benefit from programmatic malaria control measures, such as refugees	More adequately powered clinical trials in refugee camps
Garba et al.^40^	2025	Somalia	Internally displaced peoples’ camps	Prevalence and intensity of intestinal and soil-transmitted helminth infection among children	Dirty fingernails, eating unwashed fruits and walking barefoot are all not associated with increased risk of infection, including sanitary conditions at home	Improving diagnostics
Hijawi et al.^41^	2019	Jordan	Syrian refugees and Jordanian hosting communities	Detection, genotyping and phylogenetic analysis of *Leishmania* isolates	There is a high risk that species and strains might be introduced from Syria to Jordan	Comprehensive studies to confirm if any new *Leishmania tropica* strains might be introduced to Jordan by Syrian refugees
Karamitros et al.^42^	2017	Greece	Refugee camp	Enterobiasis presenting as post-traumatic stress disorder	Enterobiasis has an extremely high prevalence in overcrowded conditions	Improvement of hygienic measures
Kolimenakis et al.^43^	2022	Greece	Refugee camps	Knowledge, attitudes and perception of mosquito control	The study highlights disparities in the levels of knowledge for certain public health issues	Preparedness
Lu et al.^44^	2024	Pakistan	Hosting populations	This study aimed to scrutinize the prevalence, spatial distribution and sociodemographic and behavioural risk factors associated with cutaneous leishmaniasis in children ages <5–15 years	Afghan refugee camps in the village/district, frequent use of mosquito bed nets, presence of domestic animals in the house, knowledge of the transmission period and peak month of leishmaniasis infection increased the risk of acquiring cutaneous leishmaniasis. Refugees are predominantly susceptible and at greater risk of acquiring cutaneous leishmaniasis	The infection likely extended to new foci in the province. Monitoring is crucial
Maia et al.^45^	2018	Pakistan	Refugees	To assess the impact of topical repellents, insecticide-treated clothing and spatial repellents on malaria transmission	There is insufficient evidence to conclude topical or spatial repellents can prevent malaria	Better designed trials to generate higher certainty of evidence before well-informed recommendations can be made
Medgyesi et al.^46^	2018	Haiti	Internally displaced communities	Young child exposure to environmental hazards during play in public areas	Hand-to-mouth contact was one of the most common behaviours observed. Children touched and mouthed plastic, metal and glass trash, food and other objects from the ground, ate soil (geophagia) and drank surface water. They also touched latrines, animals, animal faeces and open drainage canals	Water, sanitation and hygiene interventions
Morseth et al.^47^	2022	Algeria	Saharawi refugee camps	To access the association between iodine concentrations in locally produced animal milk and in animal drinking water and to assess the risk of exceeding the European Food Safety Authority (EFSA) tolerable upper intake level for iodine (600 μg/day) among Saharawi women	The iodine content in goat/sheep milk was positively associated with iodine content in animal drinking water. In conclusion, consumption of camel milk and living in camps with high water iodine content increased the risk of exceeding the EFSA upper intake level for iodine	Purified water for both human and animal consumption should be provided
Mumin et al.^48^	2022	Somalia	Internally displaced persons	COVID-19 prevention: impact on livelihood, food security and mental health	COVID-19 and associated restrictions have negatively impacted Somalia’s internally displaced population	Livelihood and mental health support in the recovery phase and in future pandemic planning
Oboth et al.^49^	2019	Uganda	Refugee camps	*Plasmodium falciparum* and intestinal parasitic infections among children	*P. falciparum* and *Taenia* spp. coinfections are a stronger predictor of malaria and anaemia. The prevalence of malaria and anaemia remains higher than in the other regions in Uganda outside restricted settlements	Pragmatic intervention programs to reduce burden of coinfections
O’Connell et al.^50^	2018	Thailand	Myanmar refugees	*Ancylostoma ceylanicum* hookworm	Higher prevalence in younger age groups	Mapping to understand the distribution
Osthoff et al.^51^	2016	Switzerland	Refugees from Africa	Louse-borne relapsing fever (LBRF)	Ongoing transmission of LBRF in migrants from East Africa	Diagnosis of LBRF cases and prevention of autochthonous transmission
Patamia et al.^52^	2017	Italy	Immigrants from Africa	Geo-helminthiasis among migrants	*Ancylostomatidae, Trichuris trichiura* and *Taenia* species are the most common	Further studies in larger samples to better estimate the real burden of *Taenia solium* carriers
Seilmair et al.^53^	2016	Germany	Immigrants from Africa	Louse-borne relapsing-fever (LBRF)	It seems that our patients belong to a cluster that probably has its origin in Libya. LBRF might cause outbreaks in refugee camps	Preparedness to manage this emerging infectious disease in refugees from East Africa
Spiegel et al.^54^	2019	Kenya, Uganda, Tanzania	Nomadic communities	The socio-economic factors surrounding the initial emergence of peste des petits ruminants	Refugee movements, drought and civil unrest resulted in increased animal mingling, movement and density. Refugee camps with animal-source food demands and a robust informal economy further added to the development of animal mingling and movement as well. Once introduced, common pastoral migration lands and trade routes likely transported the disease throughout the region	Trade routes, refugee camps and areas of animal crowding during droughts should be targeted for interventions, monitoring and surveillance as part of peste des petits ruminants control in a region
Takahashi et al.^55^	2020	Jordan	Refugee camps	Cutaneous leishmaniasis vector (*Phlebotomus papatasi*)	Cutaneous leishmaniasis is endemic in Jordan yet there is a lack of robust entomological data and true reporting status is unknown. This study aimed to map habitat suitability of the main vector, *P. papatasi*	Reduced spatial target, maximizing the efficient use of resources
Takarinda et al.^56^	2022	Zimbabwe	Refugee camps	Actors associated with a malaria outbreak	The malaria outbreak at Tongogara refugee camp re-emphasizes the role of behavioural factors in malaria transmission	Intensified health education to address human behaviours that expose residents to malaria, habitat modification and larviciding to eliminate mosquito breeding sites were recommended
Vaselek et al.^57^	2019	Serbia	Transit routes	A survey of sandflies (Diptera, Phlebotominae)	After 1968, leishmaniasis was considered to be eradicated in Serbia. The recent detection of *Leishmania infantum* in sandflies and confirmed cases of leishmaniasis in humans and dogs indicated the risk of re-emergence along an important route of displaced people	Further research and monitoring
Volpato et al.^58^	2017	Algeria and Western Sahara	Sahrawi refugees	The role of *Nucularia perrinii* Batt. (Chenopodiaceae) in the camel-based Sahrawi social-ecological system	*N. perrinii* ties the ecology of the western Sahara with camel husbandry and the culture of the Sahrawi nomads	Sahrawi herders and refugees rely on the availability of this plant and tend to associate its presence with healthy camels

The majority of the studies were based in Africa (n=22), Asia (n=12) and Europe (n=9). Only one article focused on displacements in Central America. Figure 1 shows the global distribution of the studies from the perspective of the main migration routes, as described by the International Organization for Migration (IOM), confirming that all the investigations were conducted on the main routes.

**Figure 1:**
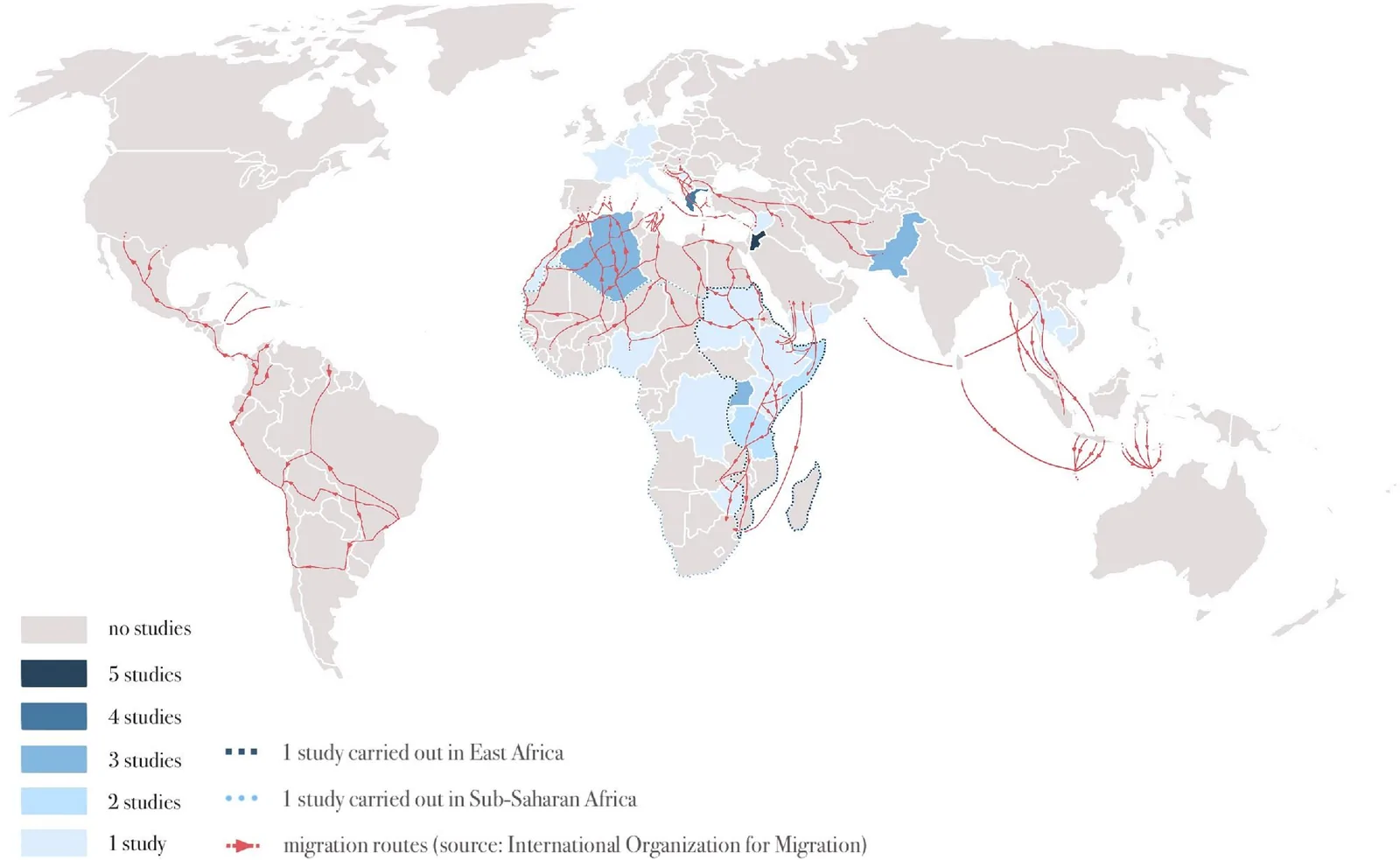
Connections between the main migration routes and the countries where the studies included in the review were carried out.

A narrative synthesis—thematically organized—highlights the main themes addressed by the peer-reviewed literature. Additionally, a summarizing table is provided (Table 2) to offer a synthetic view of the main findings from the peer-reviewed literature.

**Table 2:** Summary of the main findings.

Geographic coverage
22 studies conducted within the African Continent: Tanzania,^27,54^ sub-Saharan Africa,^28^ Congo,^29^ Nigeria,^29^ Algeria,^34,47,58^ Burundi,^34^ Rwanda,^34^ East Africa,^34^ Sudan,^35^ Ethiopia,^39^ Uganda,^39,49,54^ Somalia,^40,48^ Kenya,^54^ Zimbabwe^56^ and Western Sahara^58^
12 studies were conducted in Asia: Yemen,^25^ Jordan,^26,31,36,41,55^ Bangladesh,^30^ Syria,^32^ Cambodia,^36^ Pakistan^39,44,45^ and Thailand^50^
9 studies were conducted across Europe: France,^33^ Greece,^37,38,42,43^ Switzerland,^51^ Italy,^52^ Germany^53^ and Serbia^57^
1 study was conducted in Central America: Haiti^46^
Communities involved
Refugee camps^26–29,31–35,37–39,41–43,45,47,49–53,55–58^
Internally displaced communities^25,28,32,36,40,46,48^
Hosting communities^25,30,41,44^
Prisons^34^
Nomadic communities^54^
Homeless populations^34^
Main zoonotic, food- and vector-borne diseases
Leishmaniosis and vector-borne diseases^32,37,38,41,43,44,55,57^
Malaria and its vectors^25,27,39,45,56^
Helminths,^40,52,53^ roundworms,^33^ hookworms^50^ and intestinal parasitic coinfections^49^
Brucellosis^26^
Hepatitis E virus^28^
Monkeypox^29^
Respiratory infections^31^
Louse-borne infections^34,51^
Schistosomiasis^35^
Enterobiasis^42^
COVID-19^48^
Risk factors and areas of improvement
Environmental management and vector control^25,27,33–35,37,42,46,47^
Strengthening surveillance, monitoring and diagnostics^25,26,29,37–41,44,51^
Preparedness efforts, public health response and access to care^26,31,43,53^
Community interventions and research^28,30,36,37,45,47,48,50,52,54,56–58^

Table 3 summarizes grey literature sources that address relevant topics and emergent issues not consistently covered by peer-reviewed publications, thereby facilitating and encouraging further research. Of particular importance were the guidelines to improve the management of livestock and pet in emergencies^9,10^ and preparedness tools.^11–13^ It was essential to draw on grey literature, particularly regarding cattle camps in India,^14–23^ as this setting has not yet been documented in peer-reviewed publications and, at present, it does not appear to have been the focus of in-depth scientific investigations. This gap is notable given their critical role for local livestock-keeping communities, who are exposed to risks associated with the environmental determinants of health.

### Geographic coverage and communities involved

The studies included in this review cover a wide and diverse geographic landscape on the main migration routes (Figure 1), reflecting the global relevance of the topic and the different contexts in which affected populations are located or displaced (Table 2). Africa accounts for the largest number of articles, with 22 studies conducted across multiple regions and countries, including Tanzania,^27,54^ sub-Saharan Africa,^28^ Congo,^29^ Nigeria,^29^ Algeria,^34,47,58^ Burundi,^34^ Rwanda,^34^ East Africa,^34^ Sudan,^35^ Ethiopia,^39^ Uganda,^39,49,54^ Somalia,^40,48^ Kenya,^54^ Zimbabwe^56^ and Western Sahara.^58^

12 studies were conducted in Asia, covering a similarly diverse set of contexts. These studies examined refugees, displaced and hosting populations in Yemen,^25^ Jordan,^26,31,36,41,55^ Bangladesh,^30^ Syria,^32^ Cambodia,^36^ Pakistan^39,44,45^ and Thailand.^50^

The prominence of Jordan (five studies) and Pakistan (three studies) within this group underscores their relevant roles as major host countries for refugees and displaced communities.

Nine studies focused on refugee communities within European countries: France,^33^ Greece,^37,38,42,43^ Switzerland,^51^ Italy,^52^ Germany^53^ and Serbia.^57^ Only one study focused on internally displaced people in Central America and it was based in Haiti.^46^

Across these geographical settings, a substantial proportion of the investigations focused on refugee camps, which appear in more than two dozen studies.^26–29,31–35,37–39,41–43,45,47,49–53,55–58^

Several studies examined internally displaced communities.^25,28,32,36,40,46,48^ Other authors explored the experiences of hosting communities,^25,30,41,44^ shedding light on the social dynamics and pressures faced by local populations receiving displaced people and animals. In addition, a smaller number of studies addressed prisons,^34^ nomadic communities^54^ and homeless populations,^34^ together with refugees, illustrating the heterogeneity of vulnerable groups involved. These contexts highlight the intersection of displacement with broader social marginalization.

Displacements can disproportionately affect children, women and livestock keepers. These people are extremely vulnerable, as they can be exposed to a higher risk of zoonotic infections and violence. However, only a few studies addressed the issues experienced by children,^40,44,46,49^ women^47^ and displaced small farmers.^26,30,36,47,54,58^ These findings underscore the urgency of an integrated approach with investments in primary research to address gaps in the literature and practices, stronger policy frameworks, route-based monitoring and equity-oriented interventions.

### Main zoonotic and food- and vector-borne diseases

The studies included in the narrative review show a broad spectrum of communicable diseases affecting vulnerable populations across diverse settings, summarized in Table 2. A large number of investigations focused on vector-borne disease, particularly leishmaniasis, which appeared across multiple studies. Research addressing this and other vector-borne infections^32,37,38,41,43,44,55,57^ underscores the persistent risk posed by vectors in overcrowded environments such as refugee camps. These studies frequently point to environmental exposure, inadequate shelter and limited access to preventive measures as key drivers of transmission. Similarly, malaria and its vectors were examined in several studies,^25,27,39,45,56^ reflecting the continued burden of mosquito-borne diseases.

A significant body of work also addressed parasitic diseases, including general helminths,^40,52,53^ roundworms,^33^ hookworms^50^ and intestinal parasitic coinfections.^49^

Beyond parasitic and vector-borne diseases, the literature also identified several bacterial and viral infections of public health relevance, in particular brucellosis,^26^ hepatitis E virus^28^ and monkeypox.^29^ One study focused on respiratory infections,^31^ highlighting the ease of airborne transmission in crowded shelters and temporary housing. Louse-borne infections^34,51^ were also reported, often linked to poor hygiene conditions and limited access to laundry facilities. Additional parasitic infections such as schistosomiasis^35^ and enterobiasis^42^ were identified, each associated with specific environmental exposures and sanitation challenges.

Finally, the review captured evidence related to COVID-19.^48^

### Risk factors and areas of improvement

Across the studies included in the narrative review, a wide range of measures have been recommended to mitigate risks among displaced populations, host communities and other vulnerable groups. These recommendations span vector control, environmental health, diagnostic and surveillance capacity, public health preparedness, broader social support and research gaps to be addressed, reflecting the multifaceted nature of disease prevention in humanitarian settings (Table 2).

#### Environmental management and vector control

Vector-borne diseases require targeted interventions such as vector control, habitat modification and larvicides to reduce mosquito and sandfly populations.^27,37^ Improved living conditions, including adequate shelter and sanitation, were identified as essential to reducing exposure.^25^ Control of schistosomiasis similarly requires ongoing monitoring and environmental management.^35^ For louse-borne infections, enhanced personal hygiene measures are recommended.^34^ Many of the diseases addressed in the literature are closely linked to inadequate water, sanitation and hygiene measures.^33,42,46^ Safe water provision for humans and animals is crucial.^47^

#### Strengthening surveillance, monitoring and diagnostics

A recurring theme is the need for monitoring and robust surveillance systems to detect emerging infections early and prevent further spread.^25,44,51^ This includes the establishment of laboratory detection capacity, improved diagnostic tools and integrated disease surveillance programs.^26,29,37–41^ For zoonotic infections such as brucellosis and food-borne diseases, the literature emphasized animal vaccination, safe handling of animals and their tissues and proper preparation of dairy products.^26^

#### Preparedness efforts, public health response and access to care

Improving access to testing, vaccination and medical relief represent priorities for human and animal populations.^26,31,32,43,53^ Policies and programs involving both governments and NGOs are recommended to ensure coordinated responses.

#### Community interventions and research

Community-level strategies are essential for sustainable disease prevention, including health education to address behaviours that increase exposure to vectors or contaminated environments, diet-based interventions and awareness campaigns to improve understanding of transmission routes. Tackling food insecurity is relevant and safe animal bioproducts play an important role.^36^ Education is highlighted in particular as a key component of leishmaniasis control and malaria prevention.^37,56^ Some studies highlighted the importance of addressing broader determinants of health, including livelihood support, mental health services and community resilience during recovery phases and future pandemic planning.^30,47,48,58^ The findings consistently pointed to the need for further research to clarify transmission routes, identify sources of contamination and better estimate disease burdens.^28,45,50,52,54,57^

### Identification of gaps in holistic understanding

While peer-reviewed literature provides valuable evidence, it is still insufficient to ensure that local perspectives can be adequately represented. Guidelines, policy documents and field reports found in grey literature constitute an essential source for a holistic understanding of the topic. For this reason, this article integrated diverse forms of knowledge, like guidelines and reports from international agencies, NGOs and press coverage, summarized in Table 3.

**Table 3:** Summary of the relevant grey literature.

Setting	Source	Year	Topic	Document
Livestock-keeping during emergencies	UNHCR^9^	2005	Management of livestock in refugee and returnee situations	Practical handbook
	Practical Action Publishing^10^	2009	Livestock management during emergencies	Guidelines
(Animal) Emergency and disaster preparedness	WOAH^11^	2025	Emergency preparedness	Guidelines
	CDC^12^	2024	Pet disaster medicine	Guidelines and tools
	American Red Cross^13^	2025	Pet disaster preparedness	Guidelines and tools
	California Department of Food and Agriculture^24^	2025	Emergency support	Guidelines and tools
Indian cattle camps	Global Giving^14^	2013	Coping strategies to drought	NGO website text
	Greenpeace^15^	2016	Water storage	NGO website text
	Nashik District^16^	2019	Starting a cattle camp	Government guidelines
	Beed District^17^	2019	Information about cattle camp	Government guidelines
	Revenue & Forest Department (Relief and Rehabilitation), Government of Maharashtra^18^	2023	Disaster plan	District disaster plan (Beed)
	The Indian Express^19^	2019	Coping strategies to drought	Press article
	The New Humanitarian^20^	2019	Coping strategies to drought	Press article
	BBC News^21^	2016	Coping strategies to drought	Press article
	The Asian Age^22^	2020	Cattle camps irregularities	Press article
	Hindustan Times^23^	2019	Cattle camps and politic	Press article

In particular, guidelines on livestock keeping in contingency settings have been included.^9,10^ Also preparedness kits to address emergencies, including the aspects related to animal welfare and health issues provided by institutions like the US Centre for Disease Control and Prevention (CDC),^12^ World Organization for Animal Health (WOAH),^11^ American Red Cross^13^ and Californian Department for Food and Agriculture^24^ are essential for this review, as they strengthen its comprehensiveness.

Only one article, published in 2020 by Hussain et al.,^3^ mentioned the Indian cattle camps as a promising strategy to cope with extreme weather conditions. Since no peer-reviewed literature currently documents this setting, a search was systematically conducted through the official portals and reports of all the districts of Maharashtra—available in Hindi only^16,17^—and the Disaster Management Plan of the District of Beed, which includes them among the strategies to cope with emergencies.^18^ Furthermore, websites of NGOs reported the establishment of cattle camps in Maharashtra,^14,15^ and information about these camps can be found in online newspapers.^19–23^ These sources have been included as they raise awareness regarding settings that are currently underexplored by scientific research and advocate for multisectoral integrated approaches.

## Discussion

### General considerations

Forced displacements represent a significant challenge for both animals and their owners that must be addressed with an integrated approach. This study underscores the necessity of including companion animal and livestock management strategies in preparedness and disaster plans to strengthen global health and humanitarian responses. In particular, the role of pets and the protection of livestock health are critical components that require careful consideration.

### The role of companion animals in displacement settings

Beyond the causes of displacement—such as war, extreme environmental conditions and disasters—the species involved and their cultural role can shape how communities experience relocations. Some animals hold significant cultural meaning; for instance, singing birds help preserve the cultural identity and contribute to human well-being among Syrian refugees and for this reason are welcomed in Jordanian camps.^4^ Dogs also carry cultural roles, from family members to symbols of wealth, while in other contexts restrictions on keeping them may apply.^59^ These differences influence their destiny during displacement.

Families are sometimes unable to bring their pets due to movement restrictions or limited access of animals in refugees camps. Abandoned animals become sick, starve, die or integrate into local ecosystems.^7^ However, as pets are vital sources of emotional stability for displaced families, recognizing the human–animal bond is crucial. Integrating companion animals into emergency planning supports welfare, reduces psychological distress and strengthens global health outcomes.

### Livestock protection during conflicts

Farm animals provide essential food resources, especially in arid environments,^58^ and can serve as critical means of transport, also enhancing the chances of refugees escaping from emergencies and subsequently returning home.^4,60^

Animals migrating with their owners face challenges, including disease, stress and issues adapting to new ecological and social conditions. To minimize tensions with host communities and the risk of disease transmission, agreements and shared strategies are essential. Historical records from the early 20th century describe the management of livestock in Ottoman Mesopotamia after the World War I, where animals contributed to economic activities and biosecurity measures were implemented to control disease spread.^4^ Similar strategies have been implemented following the displacement linked to the Western Sahara conflicts in 1975 and also in 2011 in South Sudan, where refugees, hosting communities, NGOs and local authorities have been cooperating for the establishment of sustainable agreements.^6,60,61^

From a animal health perspective, cross-border livestock movement increases the risk of transboundary animal diseases. Host communities can help mitigate this risk through targeted interventions, as demonstrated in Lebanon in 2011, where emergency vaccination campaigns were implemented for livestock entering from Syria.^62^

### Promising strategies to save livestock in extreme weather conditions

Climate change and extreme weather events are ever-increasing drivers of animal displacement. In India, for instance, rising temperatures and severe droughts can cause rural–urban migrations, the depletion of farmland, a decrease in rice production and food insecurity.^63^

Maharashtra, the second most populous Indian state, regularly experiences severe droughts. During these periods, hundreds of cattle camps are established across districts to provide water, fodder and basic sanitation for livestock, as reported by the press and NGOs.^14,15,19–23^ These camps help to soften the impact of droughts and are crucial for the survival of livestock keepers’ families and NGOs have been key in documenting their relevance: for instance, Global Giving reported the establishment of a camp in 2012, with the financial support of donors and the government, enabling the provision of assistance to 6000 families from approximately 50 villages, and rescuing 15 000 cattle.^14^ Media coverage has also raised awareness of this promising strategy, reporting controversies as well.^19–23^ Thus further research is required to better understand opportunities and issues concerning Indian cattle camps as a unique promising model to overcome issues related to animal displacements. Despite their relevance, so far only one paper mentions their establishment,^3^ and to date no systematic studies have been carried out on this promising strategy, that notably has been included in the 2022–2023 Beed District Disaster Management Plan published by the government of Maharashtra to support an effective response to emergencies.^18^

### Spread of diseases at the human–animal interface

This research shows that the role of livestock in the spread of diseases within the context of the forced displacements has been investigated from a public health perspective, confirming a lack of primary data on zoonotic diseases.^2^ Research in this field is needed to raise awareness and provide solutions to potential additional issues that may arise within displaced communities and the hosting populations. The literature provided 34 papers eligible for inclusion (Table 1). The main issues reported are the spread of diseases with zoonotic potential among the displaced communities and the re/introduction of pathogens and vectors in hosting human and animal populations that may lack immunity. In particular, these studies focus on malaria and its vectors,^25,27,39,45,56^ brucellosis,^26^ hepatitis E virus,^28^ monkeypox,^29^ respiratory infections,^31^ leishmaniosis and vector-borne diseases,^32,37,38,41,43,44,55,57^ helminths,^40,52,53^ roundworms,^33^ hookworms,^50^ intestinal parasitic coinfections,^49^ louse-borne infections,^34,51^ schistosomiasis,^35^ enterobiasis^42^ and COVID-19.^48^ Peste des petits ruminants has been included in this search, even if it is not a zoonosis, as it is a highly contagious transboundary animal disease that can have a severe socio-economic impact on the income of livestock keepers.^57^ Zoonotic diseases in children have been investigated,^40,46,49^ however, there is a lack of research concerning further important vulnerable groups, for instance pregnant women and the elderly. Few papers focus on topics other than infectious diseases. One study investigated the factors contributing to the satisfaction of displaced people,^30^ one study focused on food insecurity,^36^ one focused on grazing sources for camels^58^ and one highlighted the consumption of camel and goat milk as a source of iodine.^47^ A possible higher risk of injuries or bites caused by animals in overcrowded settlements is not mentioned. There is general agreement among the authors regarding the need for further research to better investigate displacement from a global health perspective. Despite the challenges related to the implementation of preventive strategies that have been highlighted even in industrialized countries,^33^ the availability of evidence-based information could allow the improvement of a strong coordinated public health response in terms of integrated vector management,^25,27,34,37,38,44,57^ strengthened safety practices in animal handling,^26^ effective strategies to improve food safety and security,^26,36^ consolidated laboratory capability and surveillance strategies,^26,28,29,41,51–55^ sustainable vaccination campaigns,^26,29,31^ greater medical relief,^32^ better water, sanitation and hygiene facilities,^33,40,42,46,47,49,51^ improved community education,^38,44,56^ preparedness^43,48^ and policy implementation.^30^ Investments in global health research are crucial to better understand and monitor the burden of disease in displaced communities and to provide recommendations for effective, sustainable and integrated surveillance systems.

### Recommendations

#### Coping with forced animal displacements and benefits linked to the One Health approach

Animal health remains insufficiently integrated into comprehensive emergency planning, increasing the risk of disease transmission, food insecurity and conflict between displaced and host populations. International institutions already provide relevant guidance. The WOAH promotes preparedness for a wide range of biological, climatological, hydrological, geophysical and technological hazards, underscoring the importance of effective advance planning.^11^ For companion animals, preparedness guidelines from the CDC,^12^ American Red Cross^13^ and CARES^24^ highlight practical actions such as pet-inclusive evacuation planning, identification tools and the establishment of facilities capable of accommodating animals during crises. Furthermore, the UNHCR–International Union for Conservation of Nature (IUCN) livestock management handbook^9^ and practical action guidelines^10^ offer operational strategies for safely managing livestock in refugee settings.

These documents stress the need for environmental assessments, global health considerations and early coordination with host communities to manage grazing resources, water access and disease risks. Despite growing public interest in forced animal displacement, research remains limited, unevenly distributed and insufficient to inform robust policy. This study identifies the following key challenges:

- Insufficient integration of animals into national disaster plans, leaving families unprepared and animals at risk.
- Environmental pressures in displacement settings, including overgrazing and water scarcity.
- Elevated zoonotic and vector-borne disease risks due to close human–animal contact and limited health services.
- Weak cross-sector coordination between health, agriculture and environment authorities.
- Lack of research and surveillance data supporting evidence-based decision-making.

An urgent need for policy recommendation emerges from the findings of this study that can be outlined as follows:

- Integrating animal health into emergency preparedness frameworks.
- Strengthening livestock and pet management in displacement settings.
- Operationalizing One Health systems and investing in targeted research, prioritizing cost-effectiveness studies.
- Strengthening communication and awareness involving NGOs and media to highlight overlooked contexts.

A One Health approach offers a practical and scalable framework for addressing forced animal displacement. Integrating animals into preparedness planning, improving surveillance and coordination and investing in targeted research will enhance global health and strengthen resilience during crises. Cost-effectiveness studies and further research on zoonotic transmission, gender roles in livestock care and vaccination programs in refugee contexts remain essential to guide future policies.

#### Advancing health equity through a route-based approach

A growing number of conflicts and climate-driven disasters are forcing populations into temporary camps, generating complex humanitarian, environmental and global health challenges.^63^ The World Health Organization’s recent health equity report notes that 2024 saw more armed conflicts than any year since 1946, while extreme weather events continue to increase in frequency and intensity, driving dramatic increases in forced displacement and disproportionately affecting children, women and small-scale food producers.^64^ UNICEF similarly reports that millions of children are displaced annually along multidirectional migration routes.^65^ The IOM further highlights the sharp rise in the number of women and girls moving along African routes, where exposure to violence and exploitation and limited access to services is particularly high.^66^ In response, the UNHCR promotes a route-based approach to displacement governance that encompasses origin, transit and destination contexts to strengthen coordination, early warning and strategic planning.^67^ The IOM likewise argues that such an approach improves programming, capacity building and international guidance.^66^ Integrating animal health considerations into this route-based framework remains largely unexplored, despite clear intersections between human mobility and the movement of livestock and companion animals. Real-time changes in migratory patterns could be leveraged to anticipate risks related to the human–animal interface and to incorporate animal health into preventive strategies. Indeed, the studies included in this review correspond to the main movement corridors identified by the IOM^66^—including the Central, Eastern, and Western Mediterranean routes; the Balkan route; the Western and Eastern African routes; the East and Horn of Africa routes; and the Southwest and Southeast Asian routes—highlighting the need to systematically examine animal-related challenges along these pathways.

Mapping existing research on the human–animal interface along major migration routes would support knowledge sharing and help identify context-specific strategies for reducing risks linked to the displacement of animals. Intensified human–animal contact increases occupational exposure to multiple zoonotic and food-borne pathogens. Poor hygiene practices during slaughtering, butchering, handling and consuming animal products can facilitate outbreaks of diseases such as monkeypox^29^ and brucellosis,^26^ which pose elevated risks for pregnant women and foetuses. Pregnant women are also highly susceptible to hepatitis E virus,^28^ commonly transmitted through contaminated water or undercooked meat. Vector-borne diseases further threaten agricultural communities that rely on livestock and store water in open containers, which serve as breeding sites for mosquitos and other vectors.^25^ Children are particularly vulnerable: contact with animals, surface water and contaminated soil—combined with hand-to-mouth behaviour—increases the likelihood of helminth infections.^46^ High iodine levels in drinking water, reflected in animal milk, may result in excessive iodine intake,^47^ underscoring the importance of providing purified water for both humans and animals. Because women and children frequently care for animals during displacement, shelters must be located in secure areas and appropriate safeguards established to reduce exposure and prevent violence.^69^

Despite these serious challenges, displacement settings also offer opportunities to operationalize One Health through participatory, transdisciplinary cooperation. Refugee and cattle camps can function as effective platforms for implementing integrated preventive interventions targeting both human and animal health. This is particularly relevant in agricultural communities where gender roles shape exposure patterns and responsibilities for livestock care.^68^ Initiatives that address shared risks—such as improving water sanitation, providing joint vaccination campaigns, strengthening vector control and enhancing food safety practices—allow for more efficient use of scarce resources in emergency settings.^69^ Such integrated approaches maximize the potential to improve health outcomes for both populations and enable a more holistic understanding of environmental determinants of health.

Health inequalities in displacement settings stem from a complex interaction among individual, community and environmental determinants. Social gradients further exacerbate these disparities, with the most vulnerable populations experiencing the poorest outcomes.^70–72^ The WHO recommends that equity-oriented actions be central to all policies addressing forced displacement.^64^ Owczarczack-Garstecka^73^ proposes adopting the Dahlgren and Whitehead^74^ model of the social determinants of health to map hazards linked to the intensified human–animal interface in refugee camps. Applying this model would enable comprehensive situational analyses, support the identification of service gaps and guide the co-design of context-specific interventions at the individual, community and structural levels. In addition, it would strengthen the implementation of One Health by providing a systematic framework for examining the social, environmental and policy determinants that shape health outcomes among displaced populations and their animals.

Findings from this review indicate that displaced children and women with caregiving responsibilities for animals face heightened risks of zoonotic, vector-borne and food-borne diseases,^26,29,46^ as well as injury and violence.^68^ Limited access to health and veterinary services, combined with disrupted social support systems, further heightens vulnerability. Therefore, policies have to support displaced individuals in making safe behavioural choices by improving access to resources, information and services. The adoption of a health equity lens requires identifying and addressing barriers to veterinary prevention tools, diagnosis and treatments, as equitable access to these services reduces epidemic risks and protects agricultural livelihoods, which are critical to food security and economic resilience. Strengthening cooperation between displaced and host communities is essential for negotiating grazing rights, ensuring shared use of natural resources and avoiding conflicts. Embedding animal health into preparedness plans—building on existing guidelines from the WHO, WOAH, CDC and UNHCR–IUCN—will significantly contribute to improving the resilience of both human and animal populations in crisis settings.

To address these gaps, the following recommendations emerge from the literature, guidelines and field practices:

- Map and monitor main migration routes from the perspective of the human–animal interface to identify high-risk areas and inform preventive strategies.
- Incorporate strategies that improve animal health into each stage of displacement (origin, transit, destination).
- Embed One Health principles into refugee camp planning, including integrated surveillance, joint human–animal vaccination campaigns and shared vector control programs.
- Strengthen water, sanitation, food safety and security measures to reduce the transmission of food-borne and water-borne diseases, prioritizing pregnant women and children.
- Use the Dahlgren and Whitehead model to guide hazard mapping, intervention design and monitoring within a health equity framework.

Together, these recommendations articulate a coherent, evidence-based approach to managing forced animal displacement, closing current gaps and advancing integrated One Health solutions for vulnerable displaced populations and their animals.

### Limits of this narrative review

The reliance on a single freely accessible search source for peer-reviewed literature represents a limitation. Additional research on further databases could broaden the scope and capture more interdisciplinary evidence.

Furthermore, as this is a narrative review, we drew on established critical appraisal tools (such as Critical Appraisal Skills Programme checklists) as guiding frameworks to enhance methodological rigor rather than formal scoring systems. Based on these tools, we applied a set of appraisal criteria that included clarity of the research question, clarity of the methodology, appropriateness of the study design, transparency of reporting, ethical considerations and statistical robustness.

Finally, the authors recognize that grey literature does not always constitute robust evidence for scholarly review, as it often lacks the methodological rigor and peer-review standards of academic publications. Nevertheless, its inclusion in this study was necessary due to its important role in raising public awareness and documenting emerging insights on an underexplored topic that is not yet well represented in the peer-reviewed literature.

## Conclusions

Forced animal displacement is a significant but overlooked One Health challenge with direct implications for zoonotic risk, food security and social stability. This study identifies gaps in the peer-reviewed literature and highlights the importance of grey literature to achieve a comprehensive global understanding of the challenges and opportunities related to the development of effective multidisciplinary coping strategies in this setting.

Animals kept in camps contribute to the resilience of the displaced communities. Researchers and humanitarian actors should set and pursue common goals related to the human–animal interface in close cooperation with hosting communities and local authorities. We urge researchers, humanitarian agencies and policymakers to adopt a route-based, equity-oriented One Health approach to better prevent, prepare for and respond to forced animal displacements.

## Data Availability

All data are included in the article.

## References

[1] Rosinger AY, Stoler J, Ford LB et al. Mobility ideation due to water problems during historic 2022 drought associated with livestock wealth, water and food insecurity, and fingernail cortisol concentration in northern Kenya: water-related mobility ideation and stress during drought. Soc Sci Med. 2024;359:117280. 10.1016/j.socscimed.2024.117280 PMC11456390 39236480

[2] Braam DH, Jephcott FL, Wood JLN. Identifying the research gap of zoonotic disease in displacement: a systematic review. Glob Health Res Policy. 2021;6(1):25. 10.1186/s41256-021-00205-3 PMC8283393 34271977

[3] Hussain A, Memon JA, Hanif S. Weather shocks, coping strategies and farmers’ income: a case of rural areas of district Multan, Punjab. Weather Climate Extremes. 2020;30:100288.

[4] White BT . Animals, people and places in displacement. In: Adey P, Bowstead J, Brickell K et al., editors. The handbook of displacement. Cham: Palgrave McMillan; 2020:549–68.

[5] Bahta YT, Myeki VA. Adaptation, coping strategies and resilience of agricultural drought in South Africa: implication for the sustainability of livestock sector. Heliyon. 2021;7(11):e08280. 10.1016/j.heliyon.2021.e08280 PMC8571085 34765793

[6] White BT . Humans and animals in refugee camps. Forced Migr Rev. 2018;58:1–16.

[7] Angeloni G, Carr J. Animal and human health in the Sahrawi refugee camps. Forced Animal Migrations. 2018;58:80–2.

[8] Spatola GJ, Buckley RM, Dillon M et al. The dogs of Chernobyl: demographic insights into populations inhabiting the nuclear exclusion zone. Sci Adv. 2023;9(9):eade2537. 10.1126/sciadv.ade2537 PMC9984172 36867701

[9] United Nations High Commissioner for Refugees , International Union for Conservation of Nature. Livestock-keeping and animal husbandry in refugee and returnee situations a practical handbook for improved management. Geneva: United Nations High Commissioner for Refugees; 2005.

[10] LEGS . Livestock emergency guidelines and standards. Rugby, UK: Practical Action Publishing; 2009.

[11] World Organisation for Animal Health . Emergency preparedness. Available from: www.woah.org/en/what-we-offer/emergency-preparedness [accessed 28 May 2025].

[12] Centers for Disease Control and Prevention . Be prepared: pet safety in an emergency. Available from: www.cdc.gov/healthy-pets/emergency-preparedness [accessed 28 May 2025].

[13] American Red Cross . Pet disaster preparedness. Available from: https://www.redcross.org/get-help/how-to-prepare-for-emergencies/pet-disaster-preparedness.html [accessed 28 May 2025].

[14] Friends of Mann Deshi . A hope in drought: Mann Deshi Cattle Camp. Available from: https://www.globalgiving.org/projects/mann-deshi-cattle-camp [accessed 21 March 2025].

[15] Greenpeace . Water stored at a cattle camp in Maharashtra. Available from: https://media.greenpeace.org/archive/Water-Stored-at-a-Cattle-Camp-in-Maharashtra-27MZIFVE23PZ.html [accessed 21 March 2025].

[16] Nashik District . Starting Cattle Camp. Available from: https://nashik.gov.in/en/notice/starting-cattle-camp/ [accessed 21 March 2025].

[17] Beed District . Information about cattle camp 2018–2019. Available from: https://beed.gov.in/en/notice/infromation-about-cattle-camp-2018-19 [accessed 21 March 2025].

[18] Revenue & Forest Department (Relief and Rehabilitation) Government of Maharashtra . District Disaster Management Plan, Beed 2022–2023. Maharashtra, India: District Disaster Management Authority Beed; 2023.

[19] Iyer K . Maharashtra drought: year’s first cattle camp already home to over 7,000 animals. Indian Express 21 January 2019. Available from: https://indianexpress.com/article/india/maharashtra-drought-first-cattle-camp-already-home-animals-5546690/ [accessed 21 March 2025].

[20] de la Guardia M . In India, cattle camps are a last resort for families facing drought. The New Humanitarian 8 May 2019. Available from: https://www.thenewhumanitarian.org/photo-feature/2019/05/08/india-cattle-camps-are-last-resort-families-facing-drought [accessed 21 March 2025].

[21] Biswas S . India’s water refugees who live in cattle camps. BBC News 29 April 2016. Available from: https://www.bbc.com/news/world-asia-india-36147473 [accessed 21 March 2025].

[22] Shrivastava S . Probe cattle camps irregularities: Govt. The Asian Age. 31 January 2020. Available from: https://www.asianage.com/metros/mumbai/010220/probe-cattle-camps-irregularities-govt.html [accessed 21 March 2025].

[23] Gangan SP . Lok Sabha elections 2019: Maharashtra cattle camps goad villagers towards parties. Hindustan Times 6 April 2019. Available from: https://www.hindustantime.com/lok-sabha-elections/lok-sabha-elections-2019-maharsshtra-cattle-camp-goad-villagers-towards-parties [accessed 21 March 2025].

[24] California Department of Food and Agriculture . California animal response emergency support. Available from: www.cdfa.ca.gov/AHFSS/Animal_Health/eprs/cares [accessed 28 May 2025].

[25] Allan R, Weetman D, Sauskojus H et al. Confirmation of the presence of *Anopheles stephensi* among internally displaced people’s camps and host communities in Aden city, Yemen. Malar J. 2023;22:1. 10.1186/s12936-022-04427-9 PMC9806911 36593465

[26] Al-Sanouri T, Khader Y, Hailat E et al. Seroprevalence of human brucellosis among Syrian refugees in Jordan, 2022. J Pathog. 2023;2023:5885316. 10.1155/2023/5885316 PMC10748716 38146446

[27] Azizi S, Matowo J, Mbewe NJ et al. Laboratory and semi-field efficacy evaluation of permethrin–piperonyl butoxide treated blankets against pyrethroid-resistant malaria vectors. Sci Rep. 2022;12(1):22166. 10.1038/s41598-022-26804-9 PMC9774072 36550139

[28] Bagulo H, Majekodunmi AO, Welburn SC. Hepatitis E in Sub Saharan Africa – a significant emerging disease. One Health. 2020;11:100186. 10.1016/j.onehlt.2020.100186 PMC7653283 33204807

[29] Beiras CG, Malembi E, Escrig-Sarreta R et al. Concurrent outbreaks of mpox in Africa-an update. Lancet. 2024;405(10472):86–96. 10.1016/S0140-6736(24)02353-5 39674184

[30] Biswas B, Nasif Ahsan M, Mallick B. Analysis of residential satisfaction: an empirical evidence from neighbouring communities of Rohingya camps in Cox’s Bazar, Bangladesh. PLoS One. 2021;16(4):e0250838. 10.1371/journal.pone.0250838 PMC8084219 33914820

[31] Brechot C, Bryant J, Endtz H et al. 2018 international meeting of the Global Virus Network. Antiviral Res. 2019;163:140–8. 10.1016/j.antiviral.2019.01.013 PMC7127431 30690044

[32] Chambers SN, Tabor JA. Remotely identifying potential vector habitat in areas of refugee and displaced person populations due to the Syrian civil war. Geospat Health. 2018;13(2):276–80. 10.4081/gh.2018.670 30451468

[33] Claus PE, Ceuppens AS, Cool M et al. *Ascaris lumbricoides*: challenges in diagnosis, treatment and prevention strategies in a European refugee camp. Acta Clin Belg. 2018;73(6):431–4. 10.1080/17843286.2018.1436956 29458300

[34] Deng YP, Fu YT, Yao C et al. Emerging bacterial infectious diseases/pathogens vectored by human lice. Travel Med Infect Dis. 2023;55:102630. 10.1016/j.tmaid.2023.102630 37567429

[35] Elzain IA, Idris AB, Karim AA et al. Analysis of DNA *cox1* barcoding revealed novel haplotype in *Schistosoma haematobium* isolated from Western Sudan. Sci Rep. 2025;15:2062. 10.1038/s41598-025-85986-0 PMC11735769 39814875

[36] Fayemi PO, Muchenje V, Yetim H et al. Targeting the pains of food insecurity and malnutrition among internally displaced persons with nutrient synergy and analgesics in organ meat. Food Res Int. 2018;104:48–58. 10.1016/j.foodres.2016.11.038 29433783

[37] Fotakis EA, Giantsis IA, Avgerinou A et al. Identification of *Leishmania* species in naturally infected sand flies from refugee camps, Greece. Emerg Infect Dis. 2019;25(2):361–4. 10.3201/eid2502.181359 PMC6346468 30346269

[38] Fotakis EA, Giantsis IA, Castells Sierra J et al. Population dynamics, pathogen detection and insecticide resistance of mosquito and sand fly in refugee camps, Greece. Infect Dis Poverty. 2020;9(1):30. 10.1186/s40249-020-0635-4 PMC7079361 32183909

[39] Gabaldón Figueira JC, Wagah MG, Adipo LB et al. Topical repellents for malaria prevention. Cochrane Database Syst Rev. 2022;2022:CD015422. 10.1002/14651858.CD015422.pub2 PMC10440788 37602418

[40] Garba B, Asowe HA, Dirie NI et al. Prevalence and intensity of intestinal and soil-transmitted helminths infection among children in internally displaced camps in Mogadishu Somalia. Sci Rep. 2025;15:12697. 10.1038/s41598-025-93364-z PMC11993667 40221472

[41] Hijawi KJF, Hijjawi NS, Ibbini JH. Detection, genotyping, and phylogenetic analysis of *Leishmania* isolates collected from infected Jordanian residents and Syrian refugees who suffered from cutaneous leishmaniasis. Parasitol Res. 2019;118(3):793–805. 10.1007/s00436-019-06222-z 30729301

[42] Karamitros G, Kitsos N, Athanasopoulos F. A case of enterobiasis presenting as post-traumatic-stress-disorder (PTSD): a curious case of the infection with predominant mental health symptoms, presenting for the first time in the settings of a refugee camp. Pan Afr Med J. 2017;27:111. 10.11604/pamj.2017.27.111.12870 PMC5554648 28819532

[43] Kolimenakis A, Tsesmelis D, Richardson C et al. Knowledge, attitudes and perception of mosquito control in different citizenship regimes within and surrounding the Malakasa open accommodation refugee camp in Athens, Greece. Int J Environ Res Public Health. 2022;19(24):16900. 10.3390/ijerph192416900 PMC9779083 36554780

[44] Lu C, Khan K, Khan F et al. Epidemiology of cutaneous leishmaniasis in children of Khyber Pakhtunkhwa, Pakistan. Trop Med Int Health. 2024;29(7):633–46. 10.1111/tmi.14005 38740057

[45] Maia MF, Kliner M, Richardson M et al. Mosquito repellents for malaria prevention. Cochrane Database Syst Rev. 2018;2018:CD011595. 10.1002/14651858.CD011595.pub2 PMC5815492 29405263

[46] Medgyesi DN, Brogan JM, Sewell DK et al. Where children play: young child exposure to environmental hazards during play in public areas in a transitioning internally displaced persons community in Haiti. Int J Environ Res Public Health. 2018;15(8):1646. 10.3390/ijerph15081646 PMC6122025 30081490

[47] Morseth MS, Aakre I, Barikmo I et al. High iodine content in local animal milk and risk of exceeding EFSA upper intake level for iodine among Saharawi women. PLoS One. 2019;14(2):e0212465. 10.1371/journal.pone.0212465 PMC6377136 30768637

[48] Mumin FI, Wesonga FD, Handuleh JIM et al. COVID-19 and its prevention in internally displaced person (IDP) camps in Somalia: impact on livelihood, food security and mental health. BMC Public Health. 2022;22:2414. 10.1186/s12889-022-14878-z PMC9779940 36550473

[49] Oboth P, Gavamukulya Y, Barugahare BJ. Prevalence and clinical outcomes of *Plasmodium falciparum* and intestinal parasitic infections among children in Kiryandongo refugee camp, mid-Western Uganda: a cross sectional study. BMC Infect Dis. 2019;19:295. 10.1186/s12879-019-3939-x PMC6444856 30935405

[50] O’Connell EM, Mitchell T, Papaiakovou M et al. *Ancylostoma ceylanicum* hookworm in Myanmar refugees, Thailand, 2012–2015. Emerg Infect Dis. 2018;24(8):1472–81. 10.3201/eid2408.180280 PMC6056130 30014834

[51] Osthoff M, Schibli A, Fadini D et al. Louse-borne relapsing fever—report of four cases in Switzerland, June–December 2015. BMC Infect Dis. 2016;16:210. 10.1186/s12879-016-1541-z PMC4869252 27188655

[52] Patamia I, Nicotra P, Amodeo D et al. Geo-helminthiasis among migrants in Sicily: a possible focus for re-emerging neurocysticercosis in Europe. Neurol Sci. 2017;38(6):1105–7. 10.1007/s10072-017-2899-4 28289849

[53] Seilmaier M, Guggemos W, Wieser A et al. 25 Fälle von Läuserückfallfieber bei Flüchtlingen aus Ostafrika. Dtsch Med Wochenschr. 2016;141(14):e133–42. 10.1055/s-0042-108180 27404939

[54] Spiegel K, Havas K. The socioeconomic factors surrounding the initial emergence of peste des petits ruminants in Kenya, Uganda, and Tanzania from 2006 through 2008. Transbound Emerg Dis. 2019;66(2):627–33. 10.1111/tbed.13116 30632679

[55] Takahashi EA, Masoud L, Mukbel R et al. Modelling habitat suitability in Jordan for the cutaneous leishmaniasis vector (*Phlebotomus papatasi*) using multicriteria decision analysis. PLoS Negl Trop Dis. 2020;14(11):e0008852. 10.1371/journal.pntd.0008852 PMC7721129 33226979

[56] Takarinda KP, Nyadundu S, Govha E et al. Factors associated with a malaria outbreak at Tongogara refugee camp in Chipinge District, Zimbabwe, 2021: a case–control study. Malar J. 2022;21:94. 10.1186/s12936-022-04106-9 PMC8933855 35305666

[57] Vaselek S, Dvorak V, Hlavackova K et al. A survey of sand flies (Diptera, Phlebotominae) along recurrent transit routes in Serbia. Acta Trop. 2019;197:105063. 10.1016/j.actatropica.2019.105063 31207207

[58] Volpato G, Di Nardo A. The role of *Nucularia perrinii* Batt. (Chenopodiaceae) in the camel-based Sahrawi social-ecological system. J Ethnobiol Ethnomed. 2017;13:12. 10.1186/s13002-017-0141-3 PMC5299638 28179025

[59] Bardosh K, Sambo M, Sikana L et al. Eliminating rabies in Tanzania? Local understandings and responses to mass dog vaccination in Kilombero and Ulanga districts. PLoS Negl Trop Dis. 2014;8(6):e2935. 10.1371/journal.pntd.0002935 PMC4063706 24945697

[60] Pollock P . Working equids in refugee camps. Forced Migr Rev. 2018,58:75–6.

[61] Hoots C . The role of livestock in refugee–host community relations. Forced Migr Rev. 2018,58:71–4.

[62] Food and Agriculture Organization of the United Nations . Emergency vaccination against transboundary animal diseases in Lebanon. 2016. Available from: https://youtu.be/93l3inPrmpI

[63] International Organization for Migration Organization . World migration report 2024. Geneva: United Nations Research Institute for Social Development; 2024.

[64] World Health Organization . World report on social determinants of health equity. Geneva: World Health Organization; 2025.

[65] Dilger D . World Refugee Day: the biggest migration routes in the world. Available from: www.unicef.ch/en/blog/2024-06-19/world-refugee-day-biggest-migration-routes-word [accessed 28 May 2025].

[66] International Organization for Migration . The annual global overview of migration routes: 2024. Geneva: International Organization for Migration; 2025.

[67] UN Refugee Agency . Strengthening protection and solutions in the context of mixed movements of refugees and migrants. Available from: https://www.unhcr.org/media/strengthening-protection-and-solutions-context-mixed-movements-refugees-and-migrants-2024

[68] Alshawawreh L . Sheltering animals in refugee camps. Forced Migr Rev. 2018,58:76–8.

[69] Roger F, Olive MM, Peyre M et al. One Health atlas. Wallingford, UK: CAB International; 2025.

[70] Marmot Review . Fair society, healthy lives. 2010. Available from: https://www.instituteofhealthequity.org/resources-reports/fair-society-healthy-lives-the-marmot-review/fair-society-healthy-lives-full-report-pdf.pdf

[71] Marmot M . Global action on social determinants of health. Bull World Health Org. 2011;89(10):702. 10.2471/BLT.11.094862 PMC3209984 22084501

[72] Marmot M . Status syndrome: how social standing affects our health and longevity. London: Bloomsbury; 2005.

[72] Owczarczak-Garstecka S . Understanding risk in human-animal interactions. Forced Migr Rev. 2018,58:78–80.

[73] Dahlgren G, Whitehead M. The Dahlgren-Whitehead model of health determinants: 30 years on and still chasing rainbows. Public Health. 2021;199:20–4. 10.1016/j.puhe.2021.08.009 34534885

